# Supplementary material for: Splice-Junction-Based Mapping of Alternative Isoforms in the Human Proteome
Source: Cell Rep. Author manuscript; Available in PMC 2020 Jan 15. (PMC6961840; doi:10.1016/j.celrep.2019.11.026)
Supplement: 2 [file NIHMS1546469-supplement-2.zip › DF1/PXD006675/SmoothMuscleCells/SmoothMuscleCells_14_TOGARAM1_NLVQQDFLLQR.pdf]

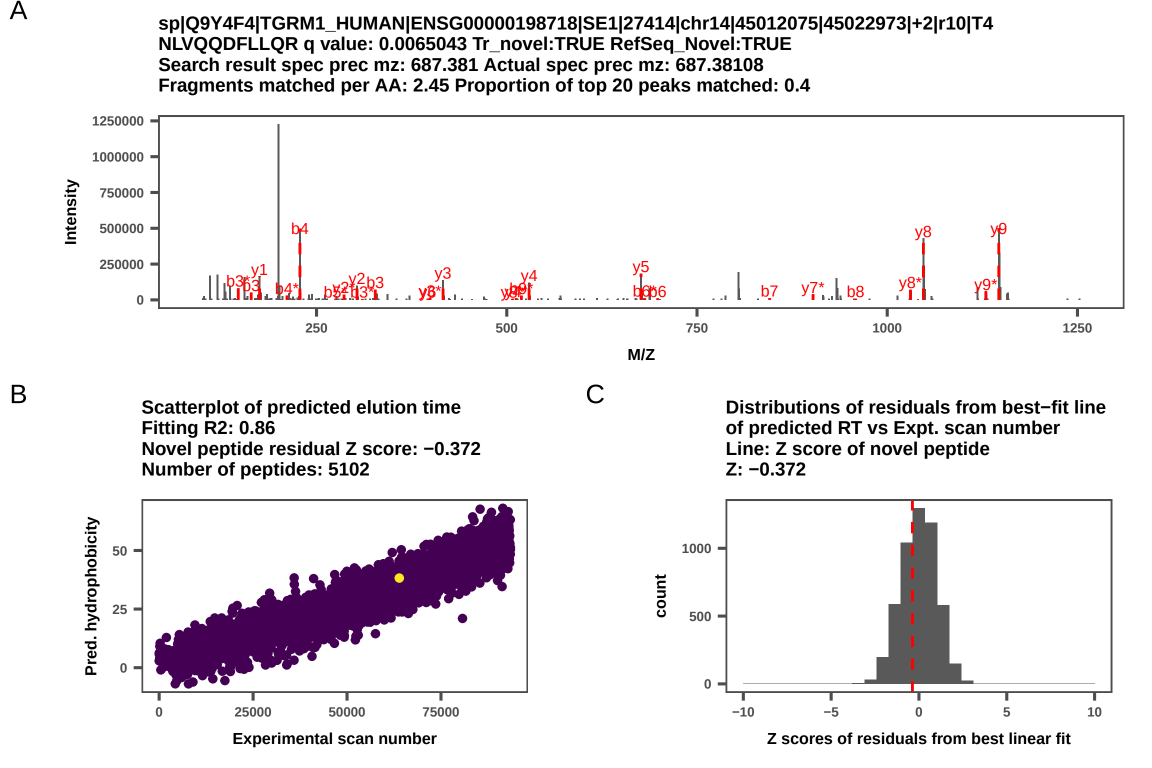
A
sp|Q9Y4F4|TGRM1_HUMAN|ENSG00000198718|SE1|27414|chr14|45012075|45022973|+2|r10|T4
NLVQQDFLLQR q value: 0.0065043 Tr_novel:TRUE RefSeq_Novel:TRUE
Search result spec prec mz: 687.381 Actual spec prec mz: 687.38108
Fragments matched per AA: 2.45 Proportion of top 20 peaks matched: 0.4
Intensity
1250000
1000000
750000
500000
250000
0
b4
y8
y9
y1
y5
y3
y4
y2
b7
b8
y7*
y8*
y9*
250
500
750
1000
1250
M/Z
B
Scatterplot of predicted elution time
Fitting R2: 0.86
Novel peptide residual Z score: −0.372
Number of peptides: 5102
Pred. hydrophobicity
50
25
0
0
25000
50000
75000
Experimental scan number
C
Distributions of residuals from best−fit line
of predicted RT vs Expt. scan number
Line: Z score of novel peptide
Z: −0.372
count
1000
500
0
−10
−5
0
5
10
Z scores of residuals from best linear fit
